# The efficacy of statins in the treatment of rheumatoid arthritis: A systematic review and meta-analysis

**DOI:** 10.1097/MD.0000000000035088

**Published:** 2023-09-15

**Authors:** Conglin Ren, Mingshuang Li

**Affiliations:** a Taizhou Hospital, Shanghai University of Traditional Chinese Medicine, Taizhou, China.

**Keywords:** DAS28, meta-analysis, RA, stains

## Abstract

**Background:**

A large body of research has investigated the use of statins in rheumatoid arthritis (RA); however, the therapeutic effects of statins remain uncertain. Thus, we designed a systematic review and meta-analysis to evaluate the role of statins in patients with RA.

**Methods:**

Databases searched to detect clinical randomized controlled trials or clinical controlled trials on the interaction between statins and RA before January 2020 included PubMed, Web of Sciences, Embase, Cochrane Library, CNKI, Wan Fang Database. Efficacy was measured by Disease Activity Score in 28 Joints (DAS28), erythrocyte sedimentation rate (ESR), C-reactive protein (CRP), tenderness of the joint (TJ), swelling of the joint (SJ), and interleukin-6. The level of blood lipid was also evaluated. STATA 12.0 was used for the meta-analysis. The Cochrane method was used for quality assessment. Heterogeneity was considered to determine fixed effects or random effects models.

**Results:**

Nineteen clinical trials with a total of 22,906 subjects were included in the meta-analysis. Sixteen studies reported a change in DAS28 after statin treatment. The pooled analysis showed that statins reduced DAS28 in RA patients. Change in ESR after statin treatment was reported in 9 studies. The summary analysis showed that statins lowered ESR in RA patients. Twelve studies reported a change in CRP after statin treatment. The results of the entire analysis showed that statins lowered CRP in RA patients. Seven studies reported a change in TJ after statin treatment. The combined analysis showed that statins reduced TJ at RA patients. Six studies reported changes in IL6 after statin therapy. The results showed that statins failed to reduce IL6 in RA patients. Seven studies reported changes in SJ after statin therapy, which showed that statins failed to reduce SJ in RA patients. We also found that statins can reduce blood lipid levels in RA patients.

**Conclusion:**

In conclusion, statins were able to reduce DAS28, ESR, CRP, TJ, and blood lipids. It indicated that stains can benefit patients with RA by inhibiting the expression of inflammatory factors and reducing the levels of lipids in the blood. Our study may offer a new perspective on the treatment of RA and provide research ideas for future larger multi-center clinical trials.

## 1. Introduction

Rheumatoid arthritis (RA), which affects more women than men, is a progressive autoimmune disease that destroys cartilage, affects joint function and reduces quality of life.^[1,2]^ Proinflammatory factors like (interleukin-6) IL-6 induce osteoclast production, promote joint damage, and cause joint swelling and degeneration.^[3]^ The cause of RA is not certain, but it is widely believed to be the result of genetic and environmental factors. It is a systemic disease with cumulative circulatory, neurological and respiratory effects, and the risk of developing adverse cardiovascular events is equivalent to that of diabetes.^[4,5]^ Early diagnosis and timely treatment of RA can help prevent damage to the joints and other organs. Currently, the main goals of RA treatment are to reduce disease activity, relieve pain, preserve joint function, and prevent systemic involvement. Disease-modifying anti-rheumatic drugs are currently the drug of choice for the treatment of RA, with a typical recommendation of about 3 months of use.^[6]^ However, due to the application of related drugs for a long time, it brings serious side effects, such as large dose dependence, kidney damage, liver enzyme abnormalities, high economic cost and etc. Hence, there was an urgent need for safe and effective medicines for RA. We were also trying to find new therapeutic strategies from a different point of view.

The efficacy of statins, also known as hmg-coa (3-hydroxy-3-methyl glutaryl coenzyme A reductase) has been well documented in preventing cardiovascular events in a variety of high-risk groups.^[7–12]^ Further research into the pathogenesis of atherosclerosis has shown that statins, in addition to lowering cholesterol levels, are able to reduce macrophage levels and attenuate the immune-inflammatory response within the plaque, thereby exerting an anti-inflammatory effect. Consequently, in addition to cardiovascular disease, statins may also be used in immune-inflammatory diseases, including RA and systemic lupus erythematosus.^[13–17]^ For clinical research data integration, machine learning and meta-analysis have become critical.^[18–20]^ Meta-analysis by Sahebkar et al^[21]^ suggests that statins were safe in treatment of systemic lupus erythematosus patients and may reduce cardiovascular risk through lowering C-reactive protein (CRP) levels.

At present, many studies have examined the effects of statins on patients with RA, but the results have been mixed. Because of the safety of statins, they might be given routinely to RA patients to help control disease activity when beneficial. We summarize high-quality, low-risk clinical trials of statins in the treatment of RA that support the efficacy of statins in the treatment of RA and provide new ideas for the therapy of RA.

## 2. Methods

### 2.1. Literature search

Databases searched included PubMed, Web of Sciences, Embase, Cochrane Library, CNKI, Wan Fang Database to identify eligible clinical trials published before January 2020. Search by the terms and medical subject headings: (“Arthritis, Rheumatoid” OR “Rheumatoid arthritis” OR “RA”) AND (“Hydroxymethylglutaryl CoA Reductase Inhibitors” OR “Inhibitors, Hydroxymethylglutaryl-CoA Reductase” OR “Statins” OR “Atorvastatin” OR “Rosuvastatin” OR “Simvastatin” OR “Pravastatin” OR “Fluvastatin” OR “lovastatin”) AND (“RCT” OR “Randomized clinical trial” OR “Clinical controlled trial” OR “CCT”). We also checked references from the reports and Internet browse.

### 2.2. Inclusion and exclusion criteria

If the following items were met, we considered the study eligible for inclusion: randomized clinical trial or clinical controlled trial; subjects were RA patients older than 18 years; interventions include statins, such as atorvastatin and simvastatin; the outcome should contain one or more targets we need. We excluded studies if any of the following items are satisfied: case reports or case series; reviews or comments. If the same author has published 2 articles with the same research in different journals, we will include the most recent one.

### 2.3. Data extraction

Two researchers independently extracted the following data from the included articles: authors’ name, publication year, country of study, trial design, number of participants in each group, characteristics of participants including age and gender, treatment of drugs and dose, length of follow-up time, duration of disease. Any discrepancies will be resolved by a third investigator.

### 2.4. Quality assessment

The quality of studies included in the analysis was assessed separately by 2 researchers using Cochrane methodology by evaluating for random sequence generation, allocation concealment, incomplete or selective outcome data reporting, blinding of participants and personnel, blinding of outcome assessment, attrition, and other sources of bias. For each domain, the risk of bias was assessed as either low, high or unclera.

### 2.5. Statistical analysis

The meta-analysis was performed using STATA version 12.0. *Z* tests were used to determine statistical differences between groups, with *P* < .05 considered statistically significant. The effect of stains in RA patients was assessed by standardized mean differences (SMDs) with 95% confidence intervals (CIs). I-square (*I*^2^) test was performed to assess the impact of study heterogeneity on the results of the meta-analysis. According to the Cochrane review guidelines, if severe heterogeneity was present at *I*^2^ > 50%, the random effect models were chosen, otherwise the fixed effect models were used. When *I*^2^ is greater than 50%, a subgroup analysis is performed to identify potential sources of heterogeneity. Moreover, we use the egger test and begg test to evaluate the publication bias. Sensitivity analysis was conducted by deleting each study individually to identify studies with high risk of bias.

## 3. Results

### 3.1. Basic characteristics and study quality

**Figure 1.**
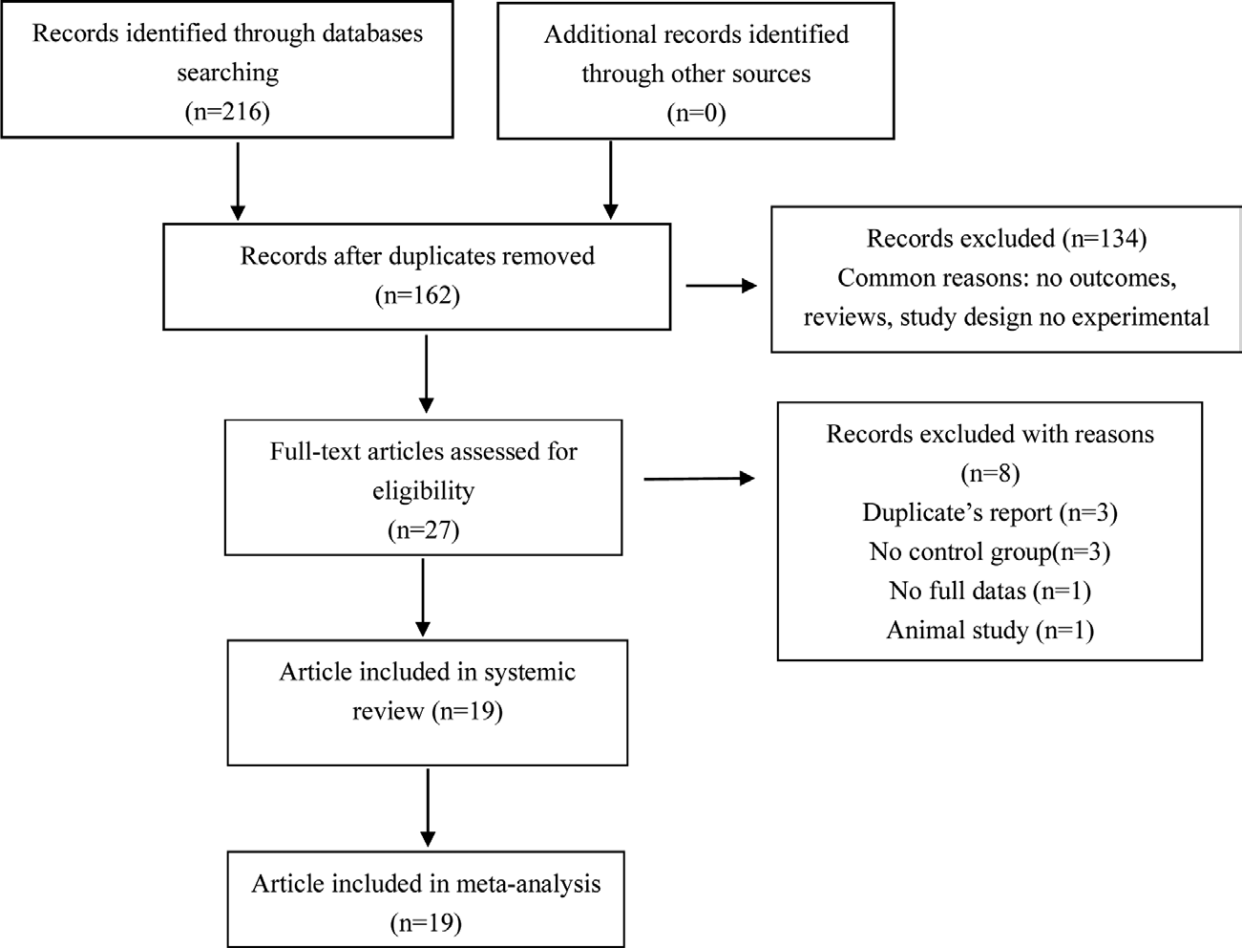
Flow diagraph of identification process for eligible studies.

**Figure 2.**
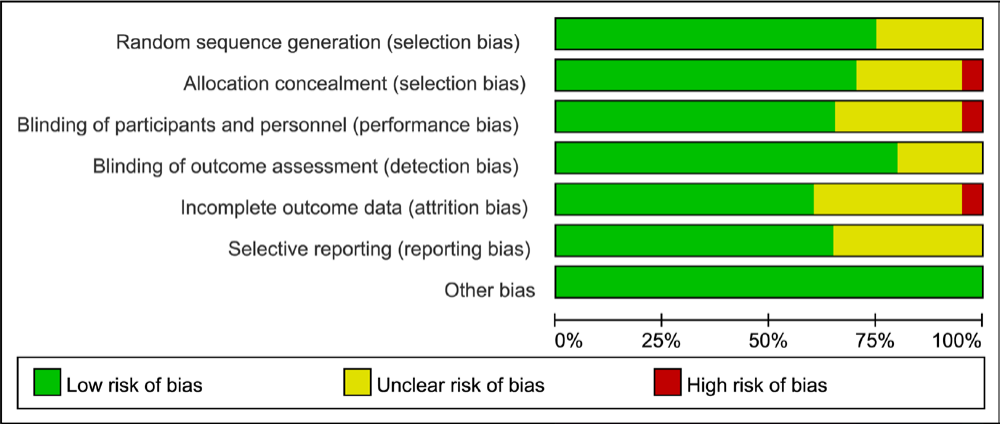
The risk of bias and quality of RCT. RCT = randomized clinical trial.

As can be seen in the flow chart (Fig. 1), we searched for a total of 216 relevant articles. After screening, 19 studies were finally included, and the basic information is shown in Table 1. In these included articles published between 2004 and 2020, a total of 22,906 patients were enrolled, with 14 studies using atorvastatin at doses ranging from 10 to 80 mg qd,^[22–35]^ 2 studies using simvastatin at doses ranging from 5 to 10 mg qd,^[36,37]^ and 3 studies using rosuvastatin at a dose of 20 mg qd.^[38–40]^ The duration of therapy ranged from 8 weeks to 12 years and most of the subjects were women. Nine of the studies were conducted in Asia, six in Europe, three in North America, and one in Africa. Figure 2 summarizes the quality risks of all studies included in the trial. Most studies were performed appropriately with randomized sequence generation and allocation concealment, and the risk of bias was low.

**Table 1:** Characteristics of 19 studies included into the meta-analysis.

Study	Years	Country	Type of statins (dosage, mg qd)	Age, yr	Participant no.	Female n, %	Duration
Ting-Ting Tang	2011	China	Atorvastatin 20	51 ± 13	55	49 (89%)	12 wk
David W	2004	UK	Atorvastatin 40	55.5 ± 11.8	116	100 (86%)	6 mo
Bansback N	2009	Canada	Atorvastatin 40	55.5 ± 11.8	116	100 (86%)	6 mo
Tam L-S	2011	China	Rosuvastatin 5–10	54 ± 5	50	37 (74%)	12 mo
Kumar P	2012	UK	Rosuvastatin 10	62.2 ± 9.65	50	37 (67%)	6 mo
George D. Kitas	2019	UK	Atorvastatin 40	61.1 ± 8.3	3002	2227 (74%)	5 yr
Amal M	2011	Egypt	Atorvastatin 20	53.7 ± 15.4	30	25 (83%)	6 mo
McInnes Iain B	2016	USA, Korea	Atorvastatin 10	51.5 ± 11.7	97	87 (90%)	12 wk
Mowla K	2016	Malaysia	Atorvastatin 40	47.8 ± 10.30	80	NA	12 wk
Semb, A G	2012	Norway	Atorvastatin 80/10	62.9 ± 8.7	18,808	NA	5 yr
Smakotina, SA	2015	Russia	Atorvastatin 20	50.9 ± 4.3	50	50 (100%)	6 mo
Cojocaru L	2013	Romania	Simvastatin 20	61.15 ± 9.49	100	86 (86%)	6 mo
CHRISTINA	2007	USA.	Atorvastatin 80	58 ± 12	20	19 (95%)	12 wk
Tikiz C	2005	Turkey	Simvastatin 20	48 ± 10	29	NA	8 wk
Sarabi ZS	2016	IRAN	Atorvastatin 40	43.1 ± 21.4	38	NA	12 wk
Mansoor Karimifar	2019	IRAN	Atorvastatin 40	46.85 ± 14.12	106	93 (88%)	6 mo
Mikhael EM	2013	Iraq	Rosuvastatin 10	43.35 ± 9.96	40	30 (75%)	8 wk
MeiYu	2011	China	Atorvastatin 20	54.3 ± 13.7	69	41 (59%)	12 wk
Harpreet Singh	2013	India	Atorvastatin 20	NA	50	NA	12 wk

### 3.2. The effect of stains according to DAS28

**Figure 3.**
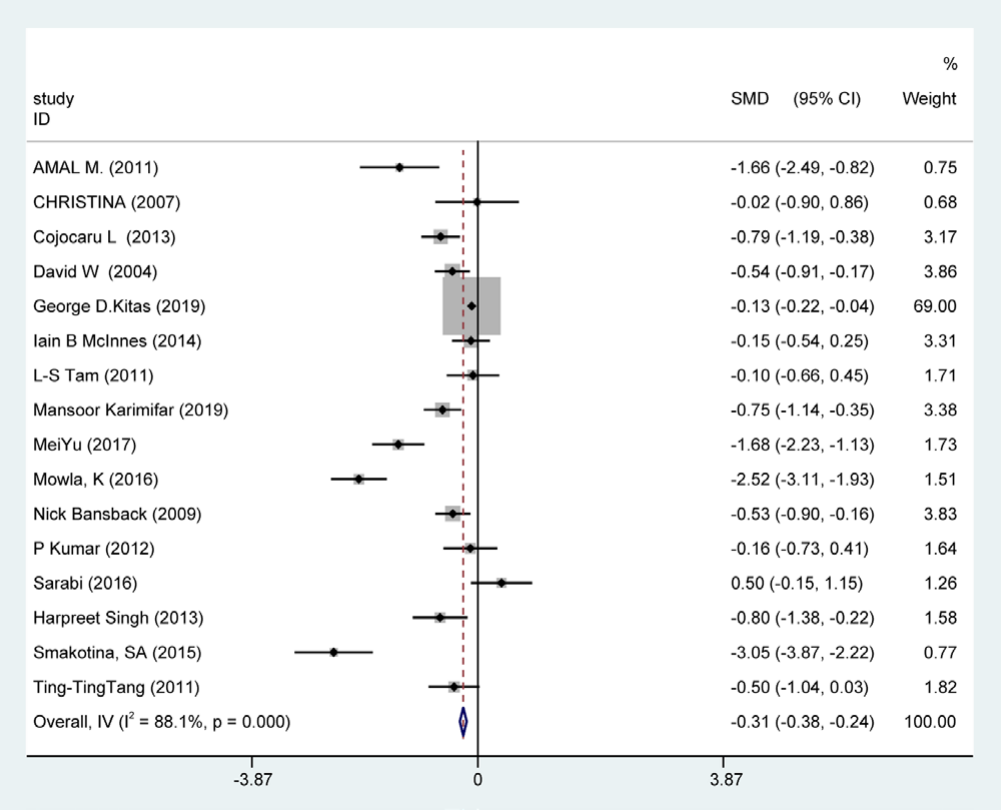
The forest graph of DAS28 in statins versus placebo. The total standardized mean difference was −0.78, 95% CI (−1.08, −0.49), *P* < .001, *I*^2^ = 88.1%. CI = confidence interval, DAS = disease activity score.

**Figure 4.**
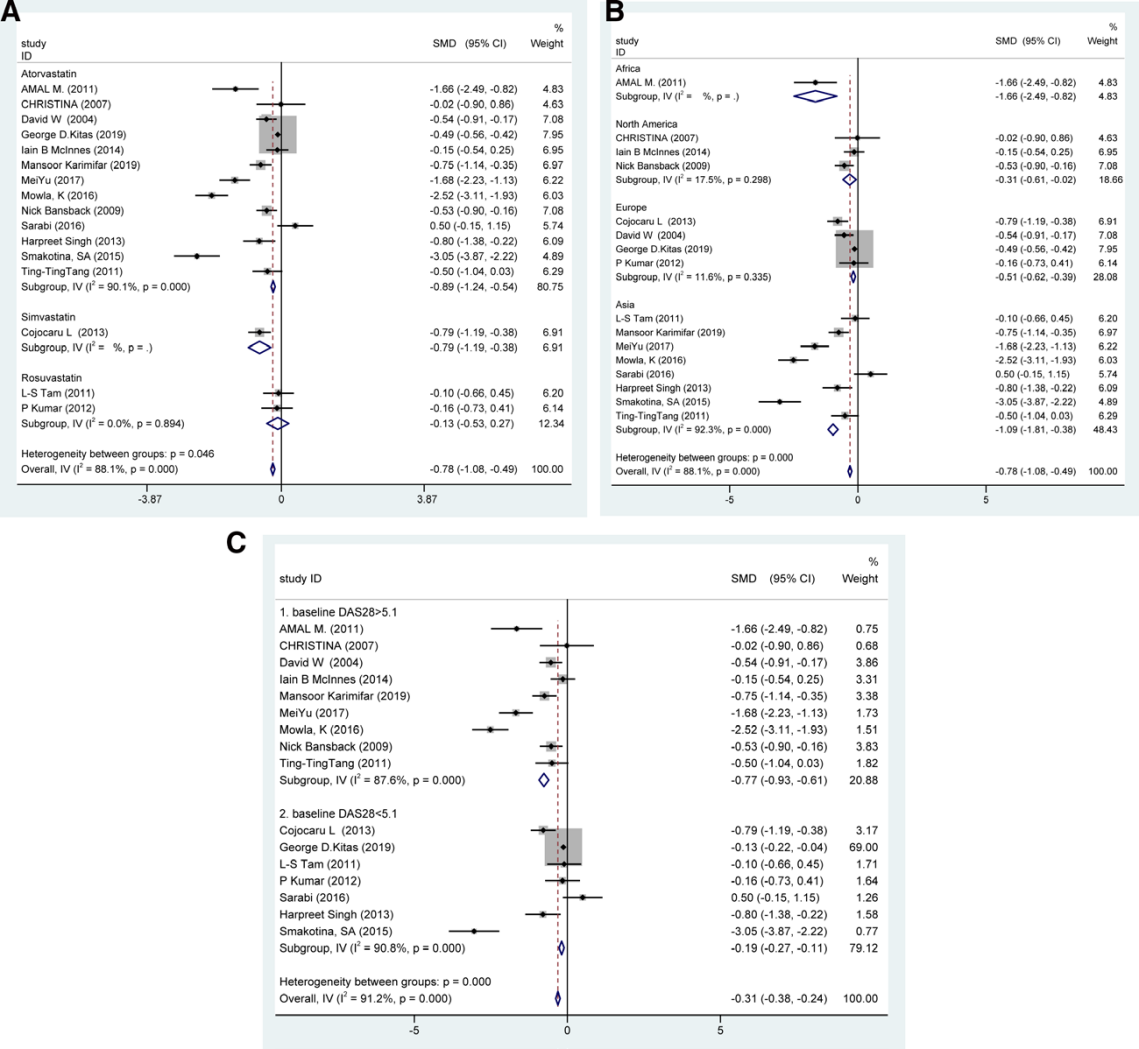
Subgroup analysis according to the type of statins (A), continent (B) and DAS28 baseline (C). (A) showed that atorvastatin significantly reduced DAS28 in RA patients (SMD −0.89, 95% CI [−1.24, −0.54], *P* < .001, *I*^2^ = 90.1%). B showed that studies from North America and Europe are less heterogeneous (*I*^2^ = 17.5% 11.6% respectively), while studies from Asia may be a source of heterogeneity (*I*^2^ = 92.3%). (C) Statin effectiveness in RA was not significantly related to baseline disease activity, and all were significantly effective. Of note, there was no change in heterogeneity regardless of the baseline DAS28. RA = rheumatoid arthritis, SMDs = standardized mean differences.

Disease Activity Score in 28 Joints (DAS28) is a composite measure of RA disease activity which includes the number of swollen and tender joints and laboratory measures such as erythrocyte sedimentation rate (ESR), and also include other general health. A total of 16 studies reported changes in DAS28 in subjects after statin treatment. It shown that statins could significantly reduce DAS28 in patients with RA [SMD = −0.785, 95% CI = −1.08, −0.49, *P* < .001] (Fig. 3). Therefore, it also found that DAS28 showed obvious heterogeneity [*I*^2^ = 86.0%, *P* = .000]. To search for sources of heterogeneity, we performed subgroup analyses based on statin type and continent. The results showed that atorvastatin significantly reduced disease activity measured based on DAS28 (SMD −0.89, 95% CI [−1.24, −0.54], *P* < .001, *I*^2^ = 90.1%). Studies from North America and Europe are less heterogeneous (*I*^2^ = 17.5%, 1.6% respectively), while studies from Asia may be a source of heterogeneity (*I*^2^ = 92.3%). Statin significantly inhibited DAS28 regardless of whether the baseline disease activity was high or low, indicating that statin has a significant effect on RA. Specifically, baseline DAS28 was not associated with heterogeneity (*I*^2^ = 87.6%, 90.8% respectively) (Fig. 4).

### 3.3. The effect of stains by other measures

Other measures including ESR, CRP, IL-6, tenderness of the joint (TJ) and swelling of the joint. The results are shown in Table 2. It can be seen that statin therapy obviously reduced CRP (SMD: −0.41, 95% CI (−0.635, −0.103), *P* = .001), ESR (SMD: −0.753, 95% CI (−0.923, −0.584), *P* = .001) and TJ (SMD: −0.402, 95% CI (−0.705, −0.098), *P* = .001). Although IL-6 (SMD: −0.167, 95% CI (−0.473, 0.139), *P* = .284) and swelling of the joint (SMD: −0.385, 95% CI (−0.926, 0.156), *P* = .163) have decreased after statin treatment, but not obvious.

**Table 2:** The effect of stains on other measures in RA patients.

	Number of trials	Number of patients	Pooled mean difference (95% CI)	*P* value	Heterogeneity *I*^2^
ESR	9	641	−0.753 (−0.923, −0.584)	.001	92%
CRP	12	3996	−0.409 (−0.635, −0.103)	.001	70.3%
IL-6	6	361	−0.167 (−0.473, 0.139)	.284	37.6%
TJ	7	397	−0.402 (−0.705, −0.098)	.01	37.5%
SJ	7	551	−0.385 (−0.926, 0.156)	.163	88.7%

CRP = C-reactive protein, ESR = erythrocyte sedimentation rate, IL-6 = interleukin-6, RA = rheumatoid arthritis, SJ = swollen joint, TJ = tender joint.

### 3.4. Changes of blood lipid level after treatment

After staining, lipid levels, including total cholesterol, triglycerides, and low-density lipoprotein cholesterol, change in RA patients. The results show that total cholesterol (SMD: −1.833, 95% CI (−2.704, −0.963), *P* = .001), triglycerides (SMD: −0.344, 95% CI (−0.530, 0.159), *P* = .001), low-density lipoprotein cholesterol (SMD: −0.544, 95% CI (−0.863, −0.393) *P* = .001) have visible decrease after treatment with statins in RA patients (Table 3).

**Table 3:** The effect of stains on blood lipids in RA patients.

	Number of trials	Number of patients	Pooled mean difference (95% CI)	*P* value	Heterogeneity *I*^2^
TC	14	23,009	−1.833 (−2.704, −0.963)	.001	99.1%
LDL-C	14	22,999	−0.544 (−0.863, −0.393)	.001	98.6%
HDL-C	14	23,108	−0.096 (−0.585, 0.393)	.701	99.5%
TG	13	22,961	−0.344 (−0.530, 0.159)	.001	94.3%

HDL-C = high density lipoprotein cholesterol, LDL-C = low-density lipoprotein cholesterol, RA = rheumatoid arthritis, TC = total cholesterol, TG = triglyceride.

### 3.5. Publication bias and analysis of sensitivity

**Figure 5.**
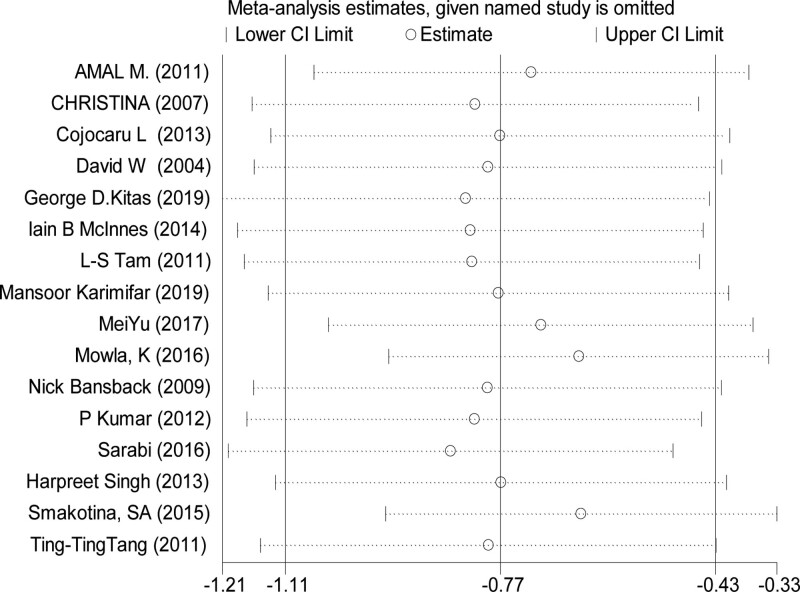
Sensitivity analysis on the effect of statins on DAS28 in RA patients. It indicated the overall effect has not changed when the studies were omitted in turn. RA = rheumatoid arthritis.

The publication bias was assessed by egger test and begg test. The results show no significant publication bias except DAS28 (Egger test *P* = .012) (Table 4). The sensitivity analysis indicated the overall effect has not changed when the studies was omitted in turn (Fig. 5).

**Table 4:** Overall analysis of publication bias on the effect of stains in RA patients.

	DAS28	ESR	CRP	TJ	SJ	IL-6
Egger test	0.012	0.525	0.361	0.437	0.380	0.191
Begg test	0.488	0.592	0.640	0.86	0.548	0.221

CRP = C-reactive protein, DAS28 = Disease Activity Score 28, ESR = erythrocyte sedimentation rate, IL-6 = interleukin-6, RA = rheumatoid arthritis, SJ = swollen joint, TJ = tender joint.

## 4. Discussion

Statin, by competitively inhibiting HMG-CoA reductase, blocking mevalonate pathway in cells, reducing cholesterol synthesis in cells, have been widely used in clinic. There is substantial evidence that statins can improve cardiac perfusion, reduce major adverse cardiac events, and reduce all-cause mortality in patients with acute coronary syndromes.^[41–43]^ Numerous studies on the use of statins in RA are currently underway. Sandooghi et al^[44]^ confirmed that atorvastatin can shorten the duration of morning stiffness in rheumatoid patients. Abdin et al^[45]^ observed the effect of statins in rats with RA, and then proved that the combination of atorvastatin and prednisolone was better than the 2 drugs alone, which could improve the lipid distribution and reduce the CRP level. Previously, Li et al^[46]^ finished a meta-analysis of the effects of statins on RA, suggesting that statins reduce DAS28 and joint swelling by inhibiting inflammation and lowering lipids, thereby improving the symptoms of RA. However, after extracting the eligible data from health insurance claims database, Lodi et al^[47]^ found that statins failed to reduce the inflammatory response of RA and to control disease progression.

This meta-analysis illustrated the effect of statins in RA patients. The results showed that statins can reduce the DAS28 score, attenuating the disease activity of RA. Statin inhibits the inflammatory response by lowering the levels of CRP and ESR, and reduces the TJ to reduce the activity of the disease, thus exerting an anti-RA effect. Simultaneously, other measures and blood lipids show a reduction in inflammation and blood lipid levels. The risk of atherosclerosis in RA patients can be increased by 2 to 3 times, and the risk of coronary heart disease, myocardial infarction, stroke and other diseases can be increased by 30% to 60%. Combined with the results of this study, we believe that statins may benefit RA patients by lowering lipids and reducing inflammation.

However, some limitations must be noticed. There was apparent heterogeneity in DAS28 based on the results of the meta-analysis. Subgroup analysis shows that studies from Asia may be the source of heterogeneity. However, it cannot be excluded that the number of studies from Asia is much larger than that from Europe and the Americas. Heterogeneity is frequent in meta-analysis. In the similar meta-analysis studied by Li et al^[46]^ they also encountered the same problem. However, the quality assessment of the literature based on the Cochrane guidelines showed that the literature included in this meta-analysis is reliable, so the conclusion is highly reliable. The Egger test showed the possibility of publication biases in trials, but no papers with publication biases were found in the sensitivity analysis. Even so, this still suggested that our research was not robust and consistent enough. Large-scale and long-term multicenter clinical trials are needed for future research. Future research content should enrich the research data based on the existing research data, focusing on the incidence of cardiovascular adverse events, the degree of Disease-modifying anti-rheumatic drugs drug dependence, and the number of NSAID applications in RA patients after statin intervention. Increasing the clinical evidence for statins in RA.

In conclusion, this systematic review and meta-analysis revealed that statins inhibit the disease activity of RA by inhibiting the expression of inflammatory factors and reducing the levels of lipids in the blood. Compared with rosuvastatin, atorvastatin inhibited DAS28 more significantly. RA patients in Asia may benefit more from statins than North America and Europe. It will offer a new perspective on the treatment of RA and provide research ideas for future larger multi-center clinical trials.

## Author contributions

**Data curation:** Conglin Ren.

**Writing – review & editing:** Conglin Ren.

**Conceptualization:** Mingshuang Li.
